# Effects of Modified Gamchogeongang-Tang on Lung Injury in a Chronic Obstructive Pulmonary Disease Mice Model: An Experimental Study

**DOI:** 10.3390/ph19010187

**Published:** 2026-01-21

**Authors:** Won-Kyung Yang, Jin Hoo Kim, Seung-Hyung Kim, Su Won Lee, In Chul Jung, Seong-Cheon Woo, Yang Chun Park

**Affiliations:** 1 Institute of Traditional Medicine and Bioscience, Daejeon University, 62 Daehak-ro, Dong-gu, Daejeon 34520, Republic of Korea; ywks1220@dju.kr (W.-K.Y.); omdhoo@naver.com (J.H.K.); tndnjs3325@daum.net (S.W.L.); wsc672@naver.com (S.-C.W.); 2 Department of Internal Medicine, College of Korean Medicine, Daejeon University, 62 Daehak-ro, Dong-gu, Daejeon 34520, Republic of Korea; sksh518@dju.kr; 3 Department of Neuropsychiatry, College of Korean Medicine, Daejeon University, 62 Daehak-ro, Dong-gu, Daejeon 34520, Republic of Korea; npjeong@dju.kr

**Keywords:** chronic obstructive pulmonary disease, lung injury, Gamchogeongang-tang, mice model, MUC5AC mRNA

## Abstract

**Objectives:** This study evaluated the effects of modified Gamchogeongang-tang (GGS01) on lung injury using a COPD mouse model. **Methods:** C57BL/6 mice were exposed to cigarette smoke extract and lipopolysaccharide and treated with GGS01 (100, 200, or 400 mg/kg). Bronchoalveolar lavage fluid (BALF) and lung tissue were analyzed using cytospin, enzyme-linked immunosorbent assay, real-time polymerase chain reaction (PCR), flow cytometry analysis, hematoxylin and eosin (H&E) and Masson’s trichrome staining, and immune histology fluorescent staining. **Results:** GGS01 significantly inhibited the increase in neutrophils in BALF, decreased immune cell activity in BALF and lung tissue, and inhibited the increase in the levels of IL-1α, TNF-α, IL-17A, MIP2, and CXCL-1 in BALF. **Conclusions:** Real-time PCR analysis showed that MUC5AC mRNA expression in lung tissue significantly decreased compared with the control group. The score of histological analysis of lung tissue damage was significantly reduced, and a decrease in IRAK1 and TNF-α expression in lung tissue was observed.

## 1. Introduction

Chronic obstructive pulmonary disease (COPD) is characterized by persistent respiratory symptoms and airflow restrictions caused by airway and parenchymal abnormalities due to exposure to smoking, surrounding (or occupational) environment, indoor air pollution, and infection [1]. Respiratory symptoms such as dyspnea and chronic cough with phlegm during exercise have been reported [2]. Moreover, COPD can exhibit various extrapulmonary effects such as reduced exercise capacity, and increased nutritional requirements and caloric intake [3]. These conditions can progress into sarcopenia, frailty, or cachexia, thereby increasing the risk of falls, hospitalizations, and death [4,5]. Treatment based on syndrome differentiation, a methodology used in oriental medicine, can potentially be used for the long-term management of COPD. Existing studies that have evaluated the effects of oriental medicine prescriptions on COPD based on syndrome differentiation classification [6] are as follows: socheongryong-tang on punghan-type [7,8]; sagan-tang [9]; sagan-tang derived SGX01 on Damtak-type [10]; maekmundong-tang [11]; saengmaekcheongpye-eum on lung deficiency-type [12]; chungsangboha-tang-derived PM014 on both lung deficiency-type and kidney yin deficiency-type [13]; gwaruhaengryeon-hwan-derived GHX02 [14]; kamkil-tang-derived GGX [15] on lung heat-type.

Modified Gamchogeongang-tang (GGS01) [16] is a prescription of Gamchogeongang-tang in combination with Jasoyeop (Haenggwanjung and Haepyosanhan) and Jinpi (Joseojung and Josuphwadam) [17]. The extract of GGS01 was prepared from authenticated raw herbs, and the yield of the extract was 22%. This has been reported as a treatment method for lung atrophy on Heohan-type in the classic clinical book of traditional Chinese medicine “Essential prescriptions from the golden cabinet treatment of causes and symptoms for coughing, lung atrophy, and abscesses.” [16]. Glycyrrhizae Radix contributes triterpene saponins (e.g., glycyrrhizin) and flavonoids (e.g., liquiritigenin). Zingiberis Rhizoma is rich in pungent principles such as gingerols and shogaols, known for their anti-inflammatory properties. Perillae Folium contains significant amounts of phenolic acids (e.g., rosmarinic acid) and flavonoids (e.g., luteolin). Citri Reticulatae Pericarpium is characterized by high levels of polymethoxyflavones and flavanone glycosides, including hesperidin and naringin. All raw materials used in GGS01 were of pharmaceutical grade, meeting the quality control standards of the Korea Food & Drug Administration (KFDA). The identity and purity of each herb were verified by expert pharmacognosists before extraction. By using verified raw materials and a fixed weight ratio, the chemical consistency of the GGS01 extract was maintained throughout the study, fulfilling the requirements for herbal pharmaceutical research [17,18].

This study investigates the effect of modified Ggamchogeongang-tang, GGS01, on COPD using an animal model of COPD induced using cigarette smoke extract (CSE) and lipopolysaccharide (LPS). Total cell counts in lung tissues and bronchoalveolar lavage fluid (BALF) were determined, and relevant cytokines were analyzed using an enzyme-linked immunosorbent assay (ELISA). The effect of this treatment on related immune cells was also determined using fluorescence-activated cell sorting (FACS). The expression level of related genes was determined using real-time polymerase chain reaction (RT-PCR). In addition, a significant effect of the treatment was confirmed through lung biopsy by evaluating its inhibitory effect on lung tissue damage. The findings of this study will help to facilitate the development of novel treatment strategies for the long-term management of COPD.

## 2. Results

### 2.1. Effect of Treatment on the Increased Number of Neutrophils in BALF

**Figure 1:**
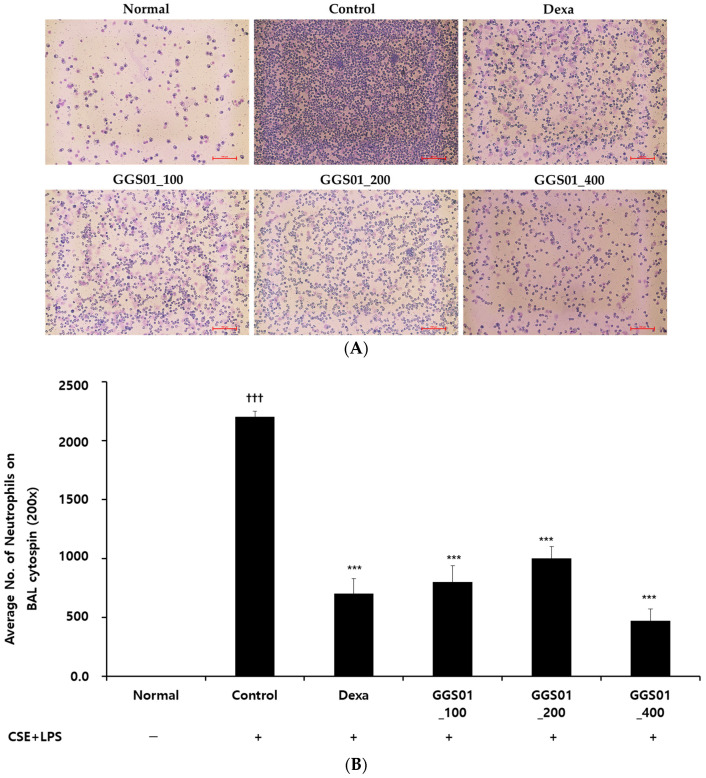
Effect of Gamchogeongang-tang (GGS01) on (**A**) cytospin image of neutrophils and (**B**) absolute number of neutrophils in the bronchoalveolar lavage fluid (BALF) of chronic obstructive pulmonary disease mice. Mice were exposed to cigarette smoke extract (CSE) + lipopolysaccharide (LPS) (control) via aspiration and then treated with dexamethasone (Dexa, 3 mg/kg) and GGS01 (100, 200, or 400 mg/kg) for 21 days (*n* = 4). Data are shown as mean ± SEM. ††† *p* < 0.001, *** *p* < 0.001.

The number of neutrophils in the COPD-induced control group was significantly increased compared with that in the normal group (2.7 ± 0.62 neutrophils), while that in the Dexa group (positive control) treated with dexamethasone after COPD inducement was 852.0 ± 140.49, 1036.0 ± 89.03, and 376.0 ± 118.11 in the experimental groups administered with 100, 200, and 400 mg/kg of GGS01, respectively, which was significantly lower compared with the control group (Figure 1).

### 2.2. Effect of Treatment on the Activity of Immune Cells in Lung Tissues

FACS analysis of the activity of immune cells in lung tissues in the COPD animal model showed a significant reduction in CD4^+^/CD3^+^ cells in the GGS01 100 mg/kg group compared with the control group. In addition, the cell numbers of neutrophils, CD8^+^/CD3^+^, CD69^+^/CD4^+^, CD8^+^/CD69^+^, CD62L−/CD44^high+^, and Gr-1^+^/siglec-F^−^ were significantly lower for all GGS01 groups compared with the control group (Table 1).

### 2.3. Effect of Treatment on Cytokine Production in BALF

**Figure 2:**
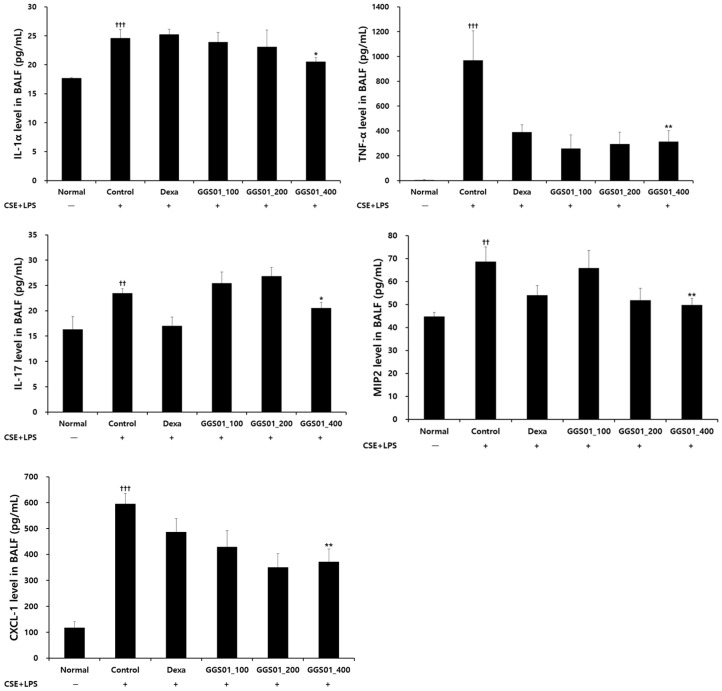
Effect of Gamchogeongang-tang (GGS01) on IL-1α, TNF-α, IL-17, MIP2, and CXCL-1 production in the bronchoalveolar lavage fluid (BALF) of chronic obstructive pulmonary disease mice. Mice were exposed to cigarette smoke extract (CSE) + lipopolysaccharide (LPS) (control) via aspiration and then treated with dexamethasone (Dexa, 3 mg/kg) and GGS01 (100, 200, or 400 mg/kg) for 21 days (*n* = 4). The level of IL-1α was determined using ELISA. Data are shown as mean ± SEM. †† *p* < 0.01. ††† *p* < 0.001,* *p* < 0.05, ** *p* < 0.01.

IL-1α level was significantly higher in the COPD-induced control than in the normal groups and significantly lower in the COPD-induced control than in the positive control and experimental groups treated with dexamethasone and GGS01 400 mg/kg. TNF-α level was higher in the control than in the normal groups. The TNF-α level was 294.03 ± 96.51 pg/mL in the experimental group treated with 200 mg/kg GGS01 and 314.54 ± 87.51 pg/mL in that treated with 400 mg/kg GGS01. This indicated that the TNF-α level was significantly lower in all experimental groups treated with GGS01 than in the control group. IL-17A level was significantly lower in the group treated with 400 mg/kg GGS01 than in the control group. The MIP2 level was significantly lower in the groups administered 200 and 400 mg/kg GGS01 than in the control group. CXCL-1 levels were 429.22 ± 62.59, 350.04 ± 52.70, and 371.98 ± 2.86 pg/mL in the experimental group treated with 100, 200, and 400 mg/kg GGS01, respectively. This indicates that the CXCL-1 level was significantly reduced in all GGS01 groups compared with that in the control group (Figure 2).

### 2.4. Effect of Treatment on MUC5CA mRNA Expression in Lung Tissues

**Figure 3:**
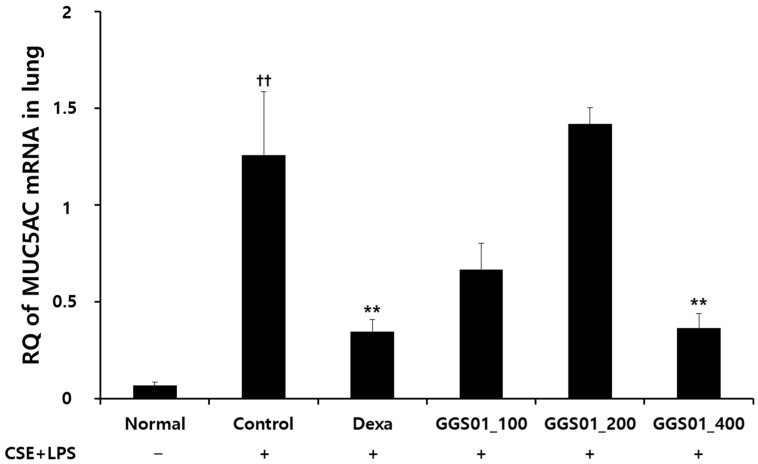
Effect of Gamchogeongang-tang (GGS01) on MUC5AC mRNA expression in the lung tissue of COPD mice. Mice were exposed to cigarette smoke extract (CSE) + lipopolysaccharide (LPS) (control) via aspiration and then treated with dexamethasone (Dexa, 3 mg/kg) and GGS01 (100, 200, or 400 mg/kg) for 21 days (*n* = 4). The level of MUC5AC was determined using real-time PCR. Data are shown as mean ± SEM. †† *p* < 0.01, ** *p* < 0.01.

MUC5AC mRNA Relative Quantitation (RQ) value was higher in the control than in the normal groups. MUC5AC mRNA RQ was 0.35 ± 0.06 in the positive control group treated with dexamethasone following COPD inducement and 0.66 ± 0.14, 1.42 ± 0.08, and 0.36 ± 0.08 pg/mL in the experimental group treated with 100, 200, and 400 mg/kg GGS01, respectively. Therefore, the MUC5AC mRNA RQ value was significantly lower in the group treated with 400 mg/kg of GGS01 than in the control group (Figure 3).

### 2.5. Effect of Treatment on Lung Tissue Damage

**Figure 4:**
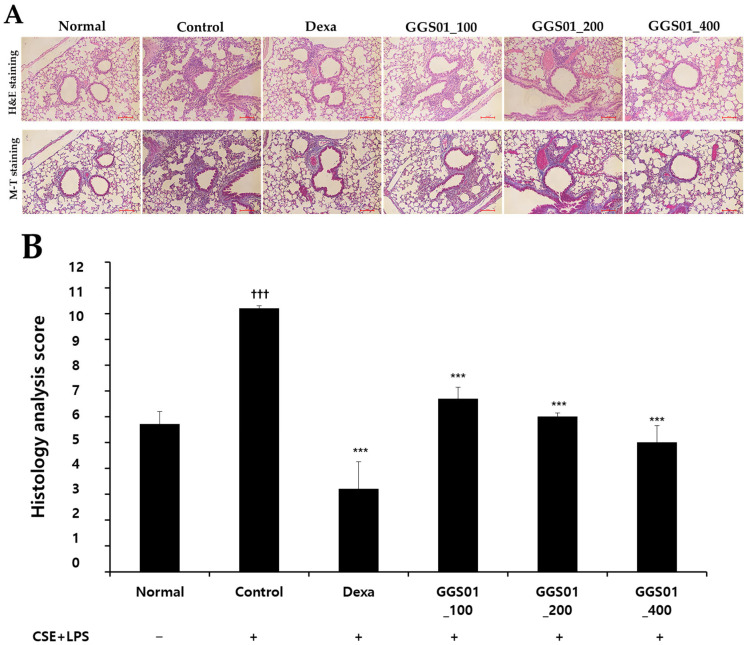
Effect of Gamchogeongang-tang (GGS01) on histopathological changes in the lung of cigarette smoke extract (CSE) + lipopolysaccharide (LPS)-induced chronic obstructive pulmonary disease mice model. Mice were exposed to CSE+LPS (control) via aspiration and then treated with dexamethasone (Dexa, 3 mg/kg) and GGS01 (100, 200, or 400 mg/kg) for 21 days (*n* = 4). (**A**) Representative sections of the lung stained with H&E stain and M-T stain (light microscope at 200× magnification). (**B**) Quantitative evaluation of the degree of lung tissue damage in the paraffin sections. Data are shown as mean ± SEM. ††† *p*<0.001, *** *p*<0.001.

**Figure 5:**
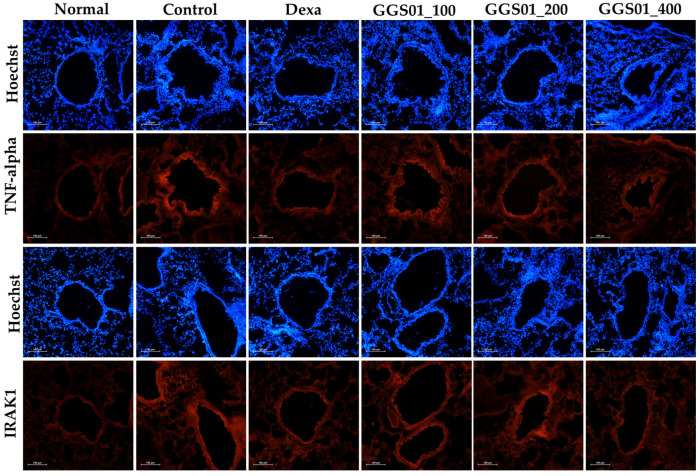
Effects of Gamchogeongang-tang (GGS01) visualized using immunohistofluorescence (IHF) staining (TNF-α and IRAK1) in the lung tissue of cigarette smoke extract (CSE) + lipopolysaccharide (LPS)-induced chronic obstructive pulmonary disease mice model.

Small and uniform alveoli were observed in the lung tissues obtained from the normal group. However, the sizes of the alveoli were not uniform, the airway walls were thickened, and the deposition of cells around the alveoli was higher in the COPD-induced control group than in the other groups. A relatively uniform alveolar shape was maintained in the positive control group treated with dexamethasone, whereas cell deposition around the alveoli was observed in the experimental groups treated with GGS01. Nevertheless, the shape of the alveoli in the experimental groups treated with GGS01 was uniform compared with that in the control group (Figure 4). When the degree of damage was quantified to evaluate the degree of lung tissue damage, the histological score of the control group was significantly higher than that of the normal group. The scores in the positive control (3.00 ± 0.00) and experimental groups treated with 100, 200, and 400 mg/kg of GGS01 were 6.50 ± 0.50, 6.00 ± 0.00, and 4.50 ± 0.50, respectively, significantly lower than those of the control group (Figure 5).

### 2.6. Effect of Treatment on the TNF-α Expression Mechanism in Lung Tissues

Regarding the expression of TNF-α and interleukin-1 receptor-associated kinase 1 (IRAK1) determined using IHF in the lung tissues of the COPD animal model, high-density nuclear staining visualized via Hoechst staining was observed in the airway periphery of the control group. An increased expression of TNF-α and IRAK1 and a decrease in the expression of TNF-α and IRAK1 were observed in the positive control and GGS01-treated groups compared with the control group (Figure 5).

### 2.7. Content of Short-Chain Fatty Acids

**Figure 6:**
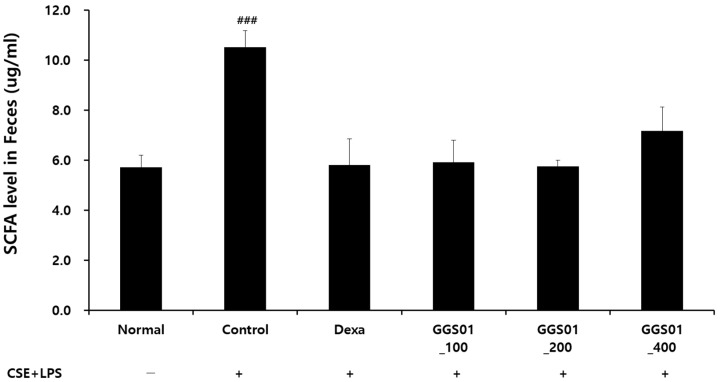
Analysis of the level of short-chain fatty acid (SCFA) in the feces of a chronic obstructive pulmonary disease-induced mouse model reflecting the gut–lung axis theory. Data are shown as mean ± SEM. ### *p* < 0.001.

The effects of GGS01 on short-chain fatty acids in the lung tissues of the COPD animal model in the small intestine production were compared (Figure 6). In the control group, compared with the normal group, the level of short-chain fatty acids in the contents of the small intestine was increased. The shortening of the contents of the small intestine fatty acid was not significantly different between the experimental groups.

## 3. Discussion

COPD is highly prevalent worldwide and associated with a high mortality rate due to exacerbation or related diseases, leading to a significant socio-economic burden and decreased quality of life [19]. The number of patients with COPD is estimated at 290 million worldwide as of 2019 [20]. In South Korea, the 2015 National Health and Nutrition Examination Survey reported that the prevalence rate in adults aged 40 years and older reached 13.4% [21]. COPD is the seventh leading cause of death worldwide as of 2017 [22] and the ninth in Korea as of 2019 [23]. In patients with COPD, structural changes occur in the airway due to repetitive injury and repair processes in each part of the lung [24]. This oxidative stress participates in the pathogenesis of COPD [25]. Proteases lead to an imbalance with anti-proteases and destroy elastin, the connective tissue in the lung parenchyma, resulting in emphysema [26]. Lymphocytes secrete various inflammatory mediators and growth factors to attract circulating inflammatory cells to the lungs and induce inflammation [27,28].

An increase in the number of activated neutrophils has been reported in the sputum or BALF of patients with COPD [29]. Macrophages promote inflammatory responses by secreting inflammatory mediators such as TNF-α, CXCL-1, CXCL-8, CCL2, and LTB_4_ [30]. CD8^+^ T lymphocytes secrete proteases to induce cell lysis and apoptosis in alveolar epithelial and vascular endothelial cells, thereby maintaining the inflammatory state [31]. Gamcho exerts an antitussive effect [32] and has shown efficacy in asthma and pneumonia animal models [33,34,35]. Geongang reduces the Th2 immune response in asthma animal models [36,37], Jasoyeop suppresses proinflammatory cytokines in lung inflammation [38], and the alkaloid in Jinpi alleviates airway constriction in guinea pigs [39]. Therefore, positive results were expected in our COPD animal model. Elastase [8,11], LPS [12,13], and cigarette smoke inhalation [40] can induce lung damage in animal models. Herein, the effect of GGS01 was evaluated following the administration of LPS and CSE, a cigarette smoke extract, into the airway of C57BL/6 mice to induce the smoking stimulus [14,15]. The number of neutrophils was significantly higher in the control group and lower in the experimental group treated with GGS01 than in the remaining groups, consistent with previous findings [10,14,15]. Elevated neutrophils promote the progression of decreased lung function due to airflow limitation in COPD patients [41] and increase airway inflammation in acute exacerbations of COPD [42]. Therefore, GGS01 may be involved in suppressing the progression of COPD and airway inflammation by reducing the number of neutrophils, which play a significant role in the pathogenesis of COPD. Mouse sialic acid-binding immunoglobulin-like lectin F (Siglec-F) is a surface receptor for eosinophils [43] used as a marker to identify neutrophils in mouse granulocytes [44]. CD11b^+^/Gr-1^+^ is a granulocyte-specific cell surface protein that induces the secretion of allergy mediators and is involved in the production of various cytokines, thereby acting as an inflammation-exacerbating factor [45]. In COPD, blood neutrophils can confirm the phenotype by the downregulated expression of CD62L [46], and CD44 plays an essential role in lymphocyte migration to inflammatory sites [47]. CD8^+^ cells are increased in asymptomatic smokers or smokers with COPD [48], suggesting that bacterial colonization may occur in the lower respiratory tract of patients with COPD [49]. CD69 is a marker for acute activation. The fraction of lung CD8^+^ T cells that express CD69 correlates with the severity of COPD [50]. The suppression of immune cell expression in lung tissues by GGS01 indicates that GGS01 can act on the immune cell-mediated pathological immune response of COPD. Herein, IL-1α, TNF-α, and IL-17A levels were significantly higher in the control group than in the normal groups and lower in the experimental group treated with GGS01 than in the control group.

IL-1α, a proinflammatory cytokine involved in the TNF-α activation pathway, plays a key role in the early stage of neutrophil-related inflammation caused by smoking and activates macrophages [51]. TNF-α level is elevated in the sputum of COPD patients [52] and serum of severe COPD patients with cachexia and skeletal muscle loss [53]. IL-17 prolongs neutrophil survival [54] and is involved in neutrophil accumulation in the peripheral airways of long-term smokers [55]. GGS01 administration suppresses the increase in MIP2 and CXCL-1 [56]. These results suggest that GGS01 suppresses the migration of inflammatory cells into the airways by reducing the expression of these chemokines. Real-time PCR analysis of lung tissues showed that the MUC5AC mRNA RQ score was significantly higher in the control than normal groups and lower in the GGS01-treated than control groups. Excessive secretion causes airway mucus obstruction, contributing to pathological conditions such as COPD [57]. GGS01 can potentially regulate airway mucus secretion in COPD by suppressing MUC5AC mRNA expression. Histological analysis showed that the alveoli of the normal group maintained a uniform shape with a constant size. However, enlarged alveoli with non-uniform shapes in the control group and collagen deposition, thickening of the airway walls, and infiltration of many inflammatory cells around the small airways were also observed. These changes reflect irreversible internal diameter reduction, such as fibrosis of the airway walls and abnormal permanent expansion of the alveoli in the small airways of <2 mm, the main locations where airflow restriction occurs in COPD [58].

## 4. Materials and Methods

### 4.1. Materials

#### 4.1.1. Drugs

The pharmaceutical ingredients of modified GGS01 used in this study were purchased from Daejeon Oriental Pharmacy (Daejeon, Republic of Korea).

The composition of the ingredients package is listed in Table 1. After adding 10-fold distilled water to 22 g of GGS01 (Table 2), extraction was performed twice at 100–120 °C for 2 h using an analog heating mantle, EAMS 9502-06 (Seoul, Republic of Korea). The obtained solutions were filtered, and the filtrate was concentrated using a rotary vacuum evaporator (Buchi B-480, Flawil, Switzerland), dried completely in a freeze dryer (Eyela FDU-540, Tokyo, Japan), and then stored at −84 °C until further use.

#### 4.1.2. Reagents and Instruments

The LPS used in this study was purchased from Sigma (St. Louis, MO, USA) and dissolved in physiological saline at a final concentration of 1 mg/mL. The solution was stored at −20 °C until further use. Mouse tumor necrosis factor-α (TNF-α, R&D systems, Minneapolis, MN, USA), mouse interleukin-6 (IL-6, R&D systems, Minneapolis, MN, USA), mouse macrophage inflammatory protein 2 (MIP2, R&D systems, Minneapolis, MN, USA), mouse chemokine (C-X-C motif) ligand-1 (CXCL-1, R&D systems, Minneapolis, MN, USA), and Fetal bovine serum (FBS) were purchased from Gibco (Thermo Fisher Scientific, Waltham, MA, USA). Dulbecco’s phosphate-buffered saline (D-PBS), formaldehyde, Dulbecco’s modified Eagle medium (DMEM), and RPMI-1640 culture medium were purchased from Sigma. The other reagents were of special grade.

Furthermore, the following equipment was used in this study: CO_2_ incubator (Forma Scientific, Marietta, OH, USA), micro-pipette (Gilson, Villiers-le-Bel, France), clean bench (Vision Scientific, Seoul, Republic of Korea), vortex mixer (Vision Scientific, Seoul, Republic of Korea), Biosystem XA (Buxco Research System, St. Paul, MN, USA), spectrophotometer (Shimadzu, Kyoto, Japan), thermocycler system (MWG Biotech, Ebersberg, Germany), deep-freezer (Sanyo, Tokyo, Japan), centrifuge (Sigma Laborzentrifugen GmbH, Osterode am Harz, Germany), plate shaker (Lab-Line, Melrose Park, IL, USA), ELISA reader (Molecular Devices, San Jose, CA, USA), and chemical balance (Cas, Seoul, Republic of Korea).

### 4.2. Methods

#### 4.2.1. Animals

Male mice C57BL/6, 7 weeks old, were purchased from Orient Bio (Seongnam, Republic of Korea) and housed in an environment with a constant temperature of 22–24 °C, humidity of 50 ± 10%, and day and night cycle (12 h day/night) adjustable lighting. The mice had ad libitum access to solid food and water. All animal protocols were approved by the Institutional Animal Care and Use Committee at Daejeon University (Approval Number: DJUARB2021-024, approved on 21 December 2021).

#### 4.2.2. Preparation of CSE

i. Cigarette combustion and smoke collection

Smoke was collected from the combustion of Coresta Monitoring Cigarette 7 (Heinr. Borgwaldt, Germany), which is used as a standard laboratory testing cigarette, in a smoking area at a relative humidity of 60 ± 5% and a temperature of 22 ± 2 °C according to the standards outlined by ISO3402 (ISO 3402 [59]; Tobacco and tobacco products—Atmosphere for conditioning and testing. International Organization for Standardization: Geneva, Switzerland, 1999). An automatic smoker RM20/CS (Heinr, Borgwaldt, Germany) was used for cigarette combustion, according to standards outlined by ISO 3308 [60]. The combustion time was set to 2.00 ± 0.02 s, wherein the length of the butt of the cigarette equaled the length of the tip paper + 3 mm.

ii. The smoking cycle was 60 ± 0.5 s, with a smoking volume of 35.0 ± 0.3 mL. A 92 mm Cambridge filter (Environmental Supply Co., Durham, NC, USA) was used to collect the smoke condensate. Extraction of the cigarette smoke condensate

The Cambridge filter, in which the cigarette smoke condensate was collected, was separated from the cigarette holder of the automatic smoking machine and placed in a 100 mL Erlenmeyer flask and 50 mL isopropanol was added. Following incubation at room temperature for >8 h, the smoke condensate was extracted, and the concentrate was filtered and concentrated using a vacuum filtration concentrator into a scintillation vial (03-340-25N, Wheaton, Millville, NJ, USA), and finally concentrated using nitrogen gas.

### 4.3. Establishment of the COPD Animal Model and Drug Administration

**Figure 7:**
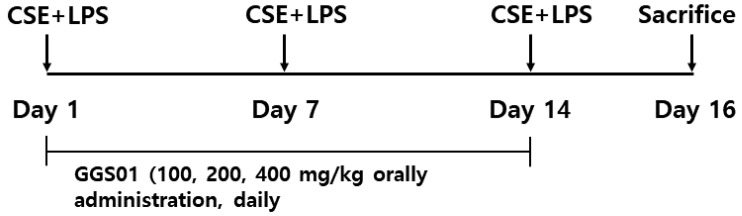
Experimental outline of cigarette smoke extract (CSE) + lipopolysaccharide (LPS) exposure. CSE+LPS: intranasal instillation of cigarette smoke extract (1 mg/mL) and LPS (100 μg/mL).

After mixing 1 mg/mL of CSE and 100 μg/mL of LPS in a 1:1 ratio, 50 μL of the mixture was aspirated into the 7-week-old C57BL/6 male mice once a week for a total of three weeks to induce COPD. The mice were anesthetized using intraperitoneal injection of 7% chloral hydrate (C8383, Sigma), and the mixture of CSE and LPS was administered through the airways. The mice were divided into experimental groups as follows: normal group (*n* = 8), no treatment; control group (*n* = 8), treated with a mixture of CSE and LPS; positive control group (Dexa, *n* = 8), orally administered 3 mg/kg of dexamethasone following treatment with the mixture of CSE and LPS; GGS01-treated groups (100, 200, and 400 mg/kg, *n* = 8 per group), orally administered GGS01 following treatment with the mixture of CSE and LPS. The drugs were administered orally daily for two weeks (Figure 7).

### 4.4. Separation of the BALF

On the last day of the experiment, the chest of mice was surgically opened to expose the airway, and a syringe was inserted into the trachea, which had been previously ligated and fixed with a string. Subsequently, a DMEM culture medium without FBS was circulated into the lung to separate the BALF. Cells isolated from BALF were treated with ammonium-chloride-potassium lysing buffer for 3 min to lyse the red blood cells and washed with DMEM. The total number of cells was determined using a hemocytometer.

### 4.5. Measurement of the Total Number of Neutrophils in BALF

Cytospin was performed to measure the number of neutrophils in BALF. Initially, precipitated blood cells were separated, and Diff-Quik staining was performed three times, following which cells were washed twice with PBS and the number of neutrophils was counted under an optical microscope (Nikon, Tokyo, Japan) at 400× magnification. Nine slides were prepared per group.

### 4.6. ELISA

An ELISA kit was used to measure the expression levels of IL-17A, TNF-α, MIP2, CXCL-1, and IL-1α in BALF. After the reaction was terminated, absorbance was measured at a wavelength of 450 nm.

### 4.7. Real-Time PCR Analysis

Real-time PCR was performed to measure the expression level of MUC5AC mRNA in the lung tissues of mice. Real-time PCR of the synthesized cDNA was performed using Applied Biosystems 7500 Fast Real-Time PCR system (Applied Biosystems, Foster City, CA, USA) and Power SYBR Green PCR Master Mix (Applied Biosystems, Foster City, CA, USA) (Table 3). A mouse glyceraldehyde-3-phosphate dehydrogenase (G3PDH) cDNA probe (Applied Biosystems, Foster City, CA, USA) was used as a control. Table 2 lists the probe sequence and primers used in this experiment. Taqman PCR Master Mix was used as a reaction solution; the final probe concentration was 200 nM. The conditions were as follows: pre-denaturation was performed at 50 °C for 2 min, 94 °C for 10 min, and 40 cycles of 95 °C for 15 s and 60 °C for 1 min. G3PDH was used as an internal standard. Relative quantitative (RQ) calculation was performed using the quantitative PCR curve y = x(1 + e)n of the target group, where y is the yield, x is the starting quantity, e is the efficiency, and n is the number of cycles.

### 4.8. Flow Cytometry Analysis of the Immune Cells

Immunofluorescence staining was performed on the isolated BALF and lung tissue cells. PE-anti-CD3e (553064), PE-anti-CD8 (553033), PE-anti-CD4 (553047), PE-anti-Gr-1 (553128), FITC Rat anti-CD69 (552879), FITC-anti-CD11b (553310), FITC Rat anti-mouse CD21/CD35 (553818), PE Rat Anti-mouse CD62L (553151), PE Rat Anti-mouse CD44 (553135), and PE-anti-Siglec-F (562068) from BD Pharmingen (San Diego, CA, USA) were used. Cell distribution was analyzed by percentage (%) using a Cell Quest program on a flow cytometer (BD Biosciences, San Diego, CA, USA). Thereafter, absolute numbers were derived based on the total number of cells.

### 4.9. Hematoxylin and Eosin (H&E) and Masson’s Trichrome (M-T) Staining

H&E and M-T staining were used to determine the degree of inflammation and blood cell infiltration in bronchioles and alveoli in relation to the lung structure to evaluate lung damage. Lung tissues were fixed in 10% neutral buffered formalin for 24 h and embedded in paraffin blocks. Slides were prepared for H&E and M-T staining and observed under an optical microscope (Nikon, Japan) at 200× magnification. The histological analysis score for lung injury was calculated by evaluating the degree of damage to bronchiole and alveolar structures, degree of infiltration of inflammatory and blood cells, and degree of collagen deposition on a three-point scale (0–2) by applying the method developed by Tanaka et al. [19].

### 4.10. Gene Expression in Lung Tissues Using Immunohistofluorescence (IHF)

Lung tissues fixed in paraffin were sectioned at a thickness of 4 µm using a cryostat (Leica, Wetzlar, Germany). The lung tissues were first incubated with anti-rabbit polyclonal filaggrin primary antibody (Abcam, Waltham, MA, USA; ab24584) and anti-rabbit FITC-conjugated IgG secondary antibody (Invitrogen, Eugene, OR, USA; R6394) and observed under a fluorescence microscope (Zeiss LSM 510, Carl Zeiss, Oberkochen, Germany).

### 4.11. Changes in Microbiota and Short-Chain Fatty Acid Levels in Feces

The colonic irrigation liquid and feces obtained at the end of the experiment were sent for analysis to AtoGen Co. (Daejeon, Republic of Korea) to evaluate the changes in microbiota according to the treatment of the complex extract of the present invention (modified GGS01) in the COPD animal model. Metagenomic DNA was extracted from fecal samples collected using the QIAamp PowerFecal Pro DNA kit (QIAGEN, Hilden, Germany) for microbiome analysis. A paired-end sequencing library was prepared by amplifying and indexing the V3-V4 regions among the nine variable regions of the 16S rRNA gene using Illumina’s Nextera XT DNA Library Prep and Nextera Index kits (San Diego, CA, USA). Sequenced data from the sequencing library were prepared using the Illumina MiSeq system. The SILVA ribosomal RNA database of v138, with a 97% similarity with QIIME2 software (version 2021.4), was used to analyze the microbiota. The results were obtained by analyzing the alpha diversity to identify the microbiome in the sample and beta diversity for the microbiota comparison between groups.

### 4.12. Statistical Analysis

Data are presented as mean ± standard error of the mean (SEM). Comparisons between groups were analyzed with independent sample *t*-tests using SPSS (version 12.0; SPSS Inc., Chicago, IL, USA). Statistical significance was set at *p*-value < 0.05 and further classified into 0.01 and 0.001.

## 5. Conclusions

In conclusion, our results confirmed that GGS01 could protect lung tissue from damage. The expression of TNF-α and IRAK1 was suppressed by GGS01 administration. IRAK1 activates the PKCα/PI3K/AKT/JNK pathway that in turn activates NF-κB, a critical component in the pathway associated with the LPS-induced expression of TNF-α [61]. Our results suggest that GGS01 inhibits TNF-α expression through a signaling pathway involving IRAK1.

## Figures and Tables

**Table 1:** Absolute number of various immune cells in chronic obstructive pulmonary disease (COPD) mouse model treated with GGS01.

Cell Phenotypes in the Lung	Normal	Control	Dexa	GGS01_100	GHX02_200	GHX02_400
Neutrophils (×10^4^ cells)	40.02 ± 4.95	343.90 ± 38.09 ^†††^	279.99 ± 23.38	201.19 ± 18.55 **	242.87 ± 5.60 **	237.96 ± 12.48 **
CD4^+^ (×10^4^ cells)	36.84 ± 5.22	128.29 ± 11.20 ^†††^	77.42 ± 3.11 ***	85.15 ± 6.79 **	111.57 ± 11.21	106.42 ± 9.21
CD8^+^ (×10^4^ cells)	15.33 ± 2.27	53.08 ± 5.41 ^†††^	43.92 ± 5.59	35.53 ± 3.52 **	41.40 ± 1.40 *	49.33 ± 7.30
CD4^+^CD69^+^ (×10^4^ cells)	1.23 ± 0.20	43.86 ± 9.21 ^†††^	11.06 ± 1.78 **	18.79 ± 2.89 **	20.58 ± 1.88 *	15.63 ± 0.91 **
CD8^+^CD69^+^ (×10^4^ cells)	0.64 ± 0.06	11.07 ± 1.46 ^†††^	4.52 ± 0.53 **	4.53 ± 0.44 ***	5.16 ± 0.72 **	4.35 ± 0.22 ***
CD62L^−^/CD44^high+^ (×10^4^ cells)	8.84 ± 0.99	129.36 ± 11.62 ^†††^	63.56 ± 1.17 ***	75.33 ± 0.30 ***	71.58 ± 2.46 ***	69.59 ± 2.04 ***
Gr-1^+^Siglec-F^−^ (×10^4^ cells)	12.85 ± 2.48	277.62 ± 30.40 ^†††^	197.73 ± 11.59 *	164.37 ± 11.24 **	163.35 ± 13.13 **	172.40 ± 32.24 *

Lung injury was induced in mice via CSE+LPS (control) aspiration and then treated with dexamethasone 3 mg/kg (Dexa) and GGS01 (100, 200, and 400 mg/kg). Data are presented as mean ± SEM (*n* = 8). ††† *p* < 0.001; * *p* < 0.05, ** *p* < 0.01, and *** *p* < 0.001.

**Table 2:** Composition of modified Gamchogeongang-tang (GGS01).

Herb	Pharmacognostic Name	Amount (g)
Gamcho	*Glycyrrhizae radix*	8.0
Geongang	*Zingiberis rhizoma*	4.0
Jasoyeop	*Perillae folium*	8.0
Jinpi	*Citri pericarpium*	2.0
Total amount		22.0

**Table 3:** Oligonucleotide sequence used for real-time polymerase chain reaction (PCR).

Gene	Primer	Sequence
MUC5AC	Forward	5′-AGAATATCTTTCAGGACCCCTGCT-3′
	Reverse	5′-ACACCAGTGCTGAGCATACTTTT-3′
G3PDH	VIC	5′-TGCATCCTGCACCACCAACTGCTTAG-3′

## Data Availability

The original contributions presented in this study are included in the article. Further inquiries can be directed to the corresponding author.
